# Diversity hotspot and unique community structure of foraminifera in the world’s deepest marine blue hole – Sansha Yongle Blue Hole

**DOI:** 10.1038/s41598-020-67221-0

**Published:** 2020-06-24

**Authors:** Qingxia Li, Yanli Lei, Raphaёl Morard, Tiegang Li, Baodong Wang

**Affiliations:** 1 Laboratory of Marine Organism Taxonomy and Phylogeny, Institute of Oceanology, Chinese Academy of Sciences, Qingdao, 266071 China; 2 Key Laboratory of Marine Sedimentology and Environmental Geology, First Institute of Oceanography, Ministry of Natural Resources, Qingdao, 266061 China; 3 MARUM Center for Marine Environmental Sciences, University of Bremen, Leobener Strasse, 28359 Bremen, Germany; 4 Pilot National Laboratory for Marine Science and Technology, Qingdao, 266237 China; 5 University of Chinese Academy of Sciences, Beijing, 100049 China; 6 Center for Ocean Mega-Science, Chinese Academy of Sciences, Qingdao, 266071 China

**Keywords:** Ecology, Molecular biology, Biogeochemistry, Ocean sciences

## Abstract

Marine blue holes are precious geological heritages with high scientific research values. Their physical and chemical characteristics are unique because of the steep-walled structure and isolated water column which create isolated ecosystems in geographically restricted areas. The Sansha Yongle Blue Hole (SYBH) is the world’s deepest marine blue hole. Here, we generated the first DNA metabarcoding dataset from SYBH sediment focusing on foraminifera, a group of protists that have colonized various marine environments. We collected sediment samples from SYBH along a depth gradient to characterize the foraminiferal diversity and compared them with the foraminiferal diversity of the costal Jiaozhou Bay (JZB) and the abyssal Northwest Pacific Ocean (NWP). We amplified the SSU rDNA of foraminifera and sequenced them with high-throughput sequencing. The results showed that the foraminiferal assemblages in SYBH were vertically structured in response to the abiotic gradients and diversity was higher than in JZB and NWP. This study illustrates the capacity of foraminifera to colonize hostile environments and shows that blue holes are natural laboratories to explore physiological innovation associated with anoxia.

## Introduction

Marine blue holes are subsurface voids that form in carbonate banks during glacial periods when sea water level is low and are submerged during interglacial periods^1^. They open directly into the present marine environment and have steep-walled structure and stratified water bodies which are characterized by oxygen deficit and high levels of H_2_S in deeper layers^2–4^ which create an isolated ecosystem. Hundreds of new species of invertebrates were described in blue holes with a large proportion of these new species being endemic to a singular blue hole^4,5^.

World-famous blue holes include Dean’s Blue Hole (202 m) in the Bahamas, Dahab Blue Hole (130 m) in Egypt and the Great Blue Hole (124 m) in Belize. The Sansha Yongle Blue Hole (SYBH), located in the South China Sea, has been explored in 2017 with a remotely operated vehicle and its depth has been estimated at ~300 m making it the deepest marine blue hole reported to date^6^. In order to characterize the biodiversity of the blue hole, we compared the foraminiferal diversity in SYBH with costal and abyssal environments.

Foraminifera are an abundant group of protists belonging to Rhizaria and a major component of marine communities. They have colonized various marine environments^7^ and freshwater bodies^8,9^. Some foraminiferal species were found to thrive in low-oxygen or even anoxic sediments^10–12^. One of their survival strategies in such environments is to use nitrate as an alternative electron acceptor to perform denitrification^13–17^. Foraminifera have a shorter life cycle compared with macrofauna and respond quickly to environmental changes such as temperature, salinity, pH and pollution levels, making them an excellent environmental indicator^18–22^. The stable isotope (δ^18^O and δ^13^C) and trace elements (mainly Mg, Ca and Sr) can be measured on fossil foraminifera to extract information about the physico-chemical condition of paleo-ocean^23–25^. These environmental and paleontological studies are exclusively based on foraminifera with a mineral shell, which belong to either the class Tubothalamea or Globothalamea, but metabarcoding surveys revealed that is the soft-shelled monothalamiids which dominate assemblages worldwide^19,21,26–29^.

In this study, we collected samples from the top of SYBH until its deepest point and complemented this sampling with a costal polluted habitat of Jiaozhou Bay (JZB) and an abyssal plains of the Northwest Pacific Ocean (NWP) for comparison. We studied the foraminiferal diversity and community structure in every locality using a metabarcoding approach. After comparing the three locations, we investigated the vertical variations of foraminiferal assemblages in SYBH and analyzed them in view of the environmental parameters of the water column.

## Results

### Data overview

**Figure 1:**
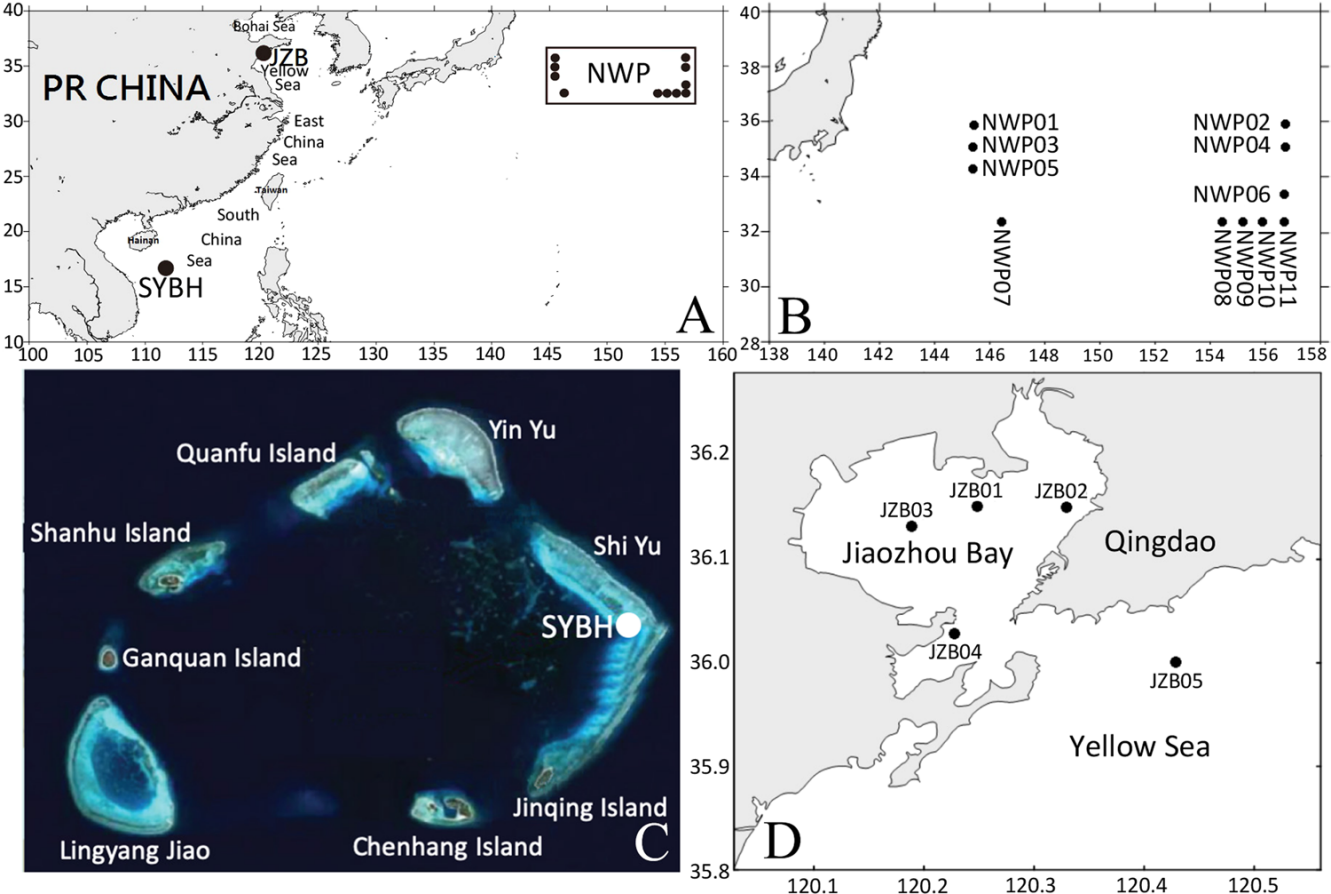
(**A**) Location of the Sansha Yongle Blue Hole (SYBH), Jiaozhou Bay (JZB) and the Northwest Pacific Ocean (NWP). Detailed sampling maps of the NWP (**B**), SYBH (**C**) and JZB (**D**).

We collected 12 sediment samples from SYBH, 5 from JZB and 11 from NWP using a combination of SCUBA diving, remotely operated vehicle (ROV), box corer and grab sampler between 2016 and 2017 (Fig. 1, Table S1). From the 28 samples, we extracted the DNA and amplified the hypervariable foraminifera specific 37 F metabarcode^19,21,28,29^ and sequenced it using Illumina HiSeq system (See Methods section). We obtained a total of 2,363,523 raw paired-end reads from the sequencing platform for the entire dataset which were spliced into 2,083,830 raw reads after assembly (Table S2). After quality filtration of raw reads, we retained 1,781,004 high-quality effective reads. After aligning all high-quality effective reads to the representative sequences of OTUs, we obtained 3,870 OTUs (Table S3). We conducted a series of strict filtering on OTUs and excluded 772 OTUs which occurred in a single sample or had an abundance of less than 10 reads or were not attributed to foraminifera by the Protist Ribosomal Reference (PR^2^) database^30^. Finally, we retained 3,098 OTUs representing 1,715,286 reads for downstream analysis (Table S3).

**Figure 2:**
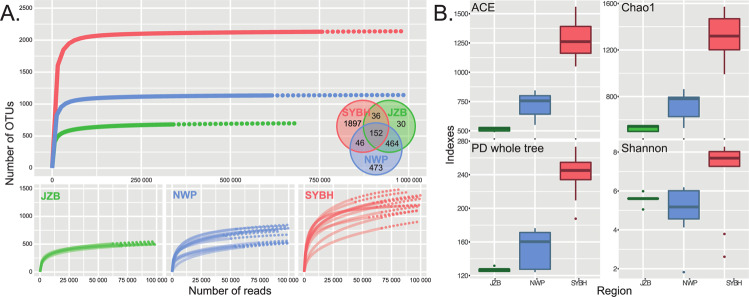
(**A**) Rarefaction curves at regional scale and at individual sample scale for JZB, NWP and SYBH, respectively calculated with the iNEXT package^57^. The solid lines were drawn based on the abundance of observed reads, and the dotted lines were drawn based on the abundance of extrapolated reads. The Venn diagram indicates the distribution of the OTUs in the three locations. (**B**) Alpha diversity indexes calculated on the normalized data for the three locations. The plot was generated with the ggplot2 package^58^.

The rarefaction curves were plotted at regional and sample level based on the number of eventually retained OTUs and reads (Fig. 2A). Saturation was reached at regional scale, in all individual samples of NWP and JZB and only one sample of SYBH. Regardless of the saturation degree of samples, the diversity of SYBH samples were systematically higher than individual samples of JZB and NWP, and at regional scale the SYBH displayed a level of diversity two times higher than the NWP and three times higher than the JZB (Fig. 2A). We observed 2131, 1135 and 682 OTUs occurring in SYBH, NWP and JZB respectively, only 152 OTUs were common in the three habitats. 464 OTUs were shared by NWP and JZB, 46 OTUs were found in both SYBH and NWP, and only 36 OTUs were present in both SYBH and JZB. The OTUs present only in one location were respectively 1897, 473 and 30 for SYBH, NWP and JZB (Fig. 2A). Four normalized alpha-diversity indexes systematically showed that SYBH had higher foraminiferal diversity than the other two locations (Fig. 2B), and the JZB was the least diversified (except for the Shannon index).

### Geographic community composition

Among 3,098 foraminiferal OTUs, 1,827 OTUs were attributed to the class Globothalamea, and 1,051 OTUs were assigned to the paraphyletic clade monothalamiids. Only 118 OTUs were assigned to the class Tubothalamea, and 102 OTUs were a part of Foraminifera-X (Table S3).

**Figure 3:**
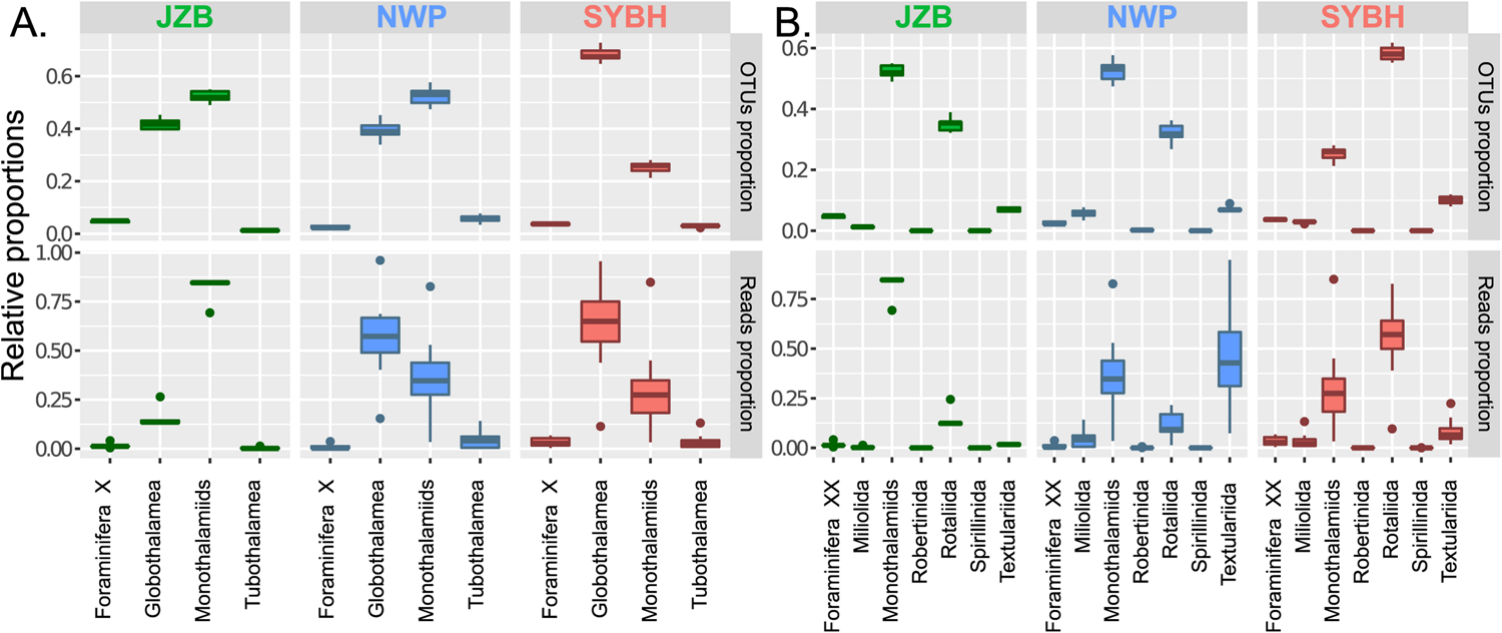
Proportions of OTUs and reads assigned to different foraminiferal classes (**A**) and orders (**B**) in three locations plotted using the ggplot2 package^58^.

At the class level, the three locations had entirely different foraminiferal community composition (Fig. 3A). In JZB, the monothalamiids occupied 80% of reads and 50% of OTUs. In NWP, about 57% of reads were assigned to the class Globothalamea, while more than 50% of OTUs represented the monothalamiids. In SYBH, over 60% of both reads and OTUs represented the class Globothalamea. At the order level, the dominant taxa in the three locations were also different (Fig. 3B). In JZB, the foraminiferal community was dominated by the soft-walled monothalamous taxa that occupied the highest proportion in both reads and OTUs. In NWP, a close proportion of reads were shared by the monothalamous taxa and the multi-chambered agglutinated Textulariida, but more than 50% of the OTUs were assigned to the monothalamous taxa and only about 6% of OTUs were assigned to Textulariida. For JZB and NWP, the multi-chambered hyaline order Rotaliida accounted for less than 15% of the reads which could represent ~30% of the OTUs. The foraminiferal composition in SYBH was markedly different, with a clear domination of the Rotaliida that accounted for more than half of the OTUs and reads volume.

**Figure 4:**
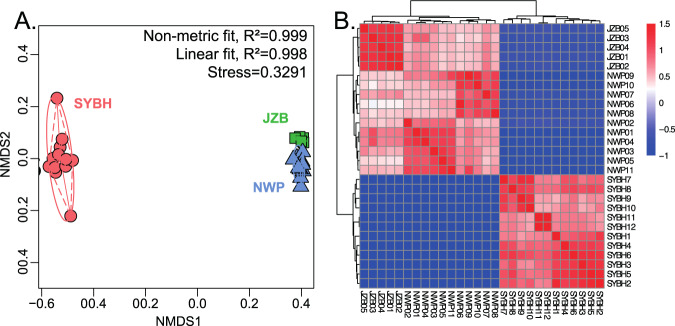
(**A**) Community structuring of benthic foraminifera using nonlinear multidimensional scaling based on Bray-Curtis distance similarity coefficient. (**B**) Heatmap of 28 samples based on the Spearman’s correlation coefficient.

This difference in community composition was clearly reflected in the Non-Metric Multi-Dimensional Scaling (NMDS) (Fig. 4A). SYBH samples were the most different of the three locations, away from JZB samples and NWP samples, while the latter two were close to each other. Multi Response Permutation Procedure (MRPP) analysis showed that the dissimilarities among the three locations were significantly greater than the dissimilarities within each location (Table 1). Heatmap of 28 samples based on the Spearman’s correlation coefficient further clarified the community structuration within the location that was not visible on the NMDS (Fig. 4B). The JZB samples had a homogeneous community whilst the NWP were divided into two clusters (NWP06, NWP07, NWP08, NWP09, NWP10 and NWP01, NWP02, NWP03, NWP04, NWP05, NWP11) which could be driven by spatial heterogeneity (except for NWP11). Twelve SYBH samples were divided into four groups: the first group (SYBH1) with a water depth of 3.1 m; the second group (SYBH2 to SYBH6) with a water depth of less than 20 m; the third group (SYBH7 to SYBH 10) with a water depth of 20–40 m; and the fourth group (SYBH11 and SYBH12) with a water depth of over 100 m.

**Table 1:** Multi Response Permutation Procedure (MRPP) analysis. The A value greater than zero indicates that the dissimilarities among groups is greater than the dissimilarities within the group; the larger the Expect delta is, the greater the dissimilarities among groups are; the *p* value of less than 0.05 means a significant difference.

group	A	Observe delta	Expect delta	*p* value
SYBH-JZB	0.1341	0.7608	0.8785	0.002
SYBH-NWP	0.1859	0.6943	0.8528	0.001
JZB-NWP	0.1411	0.5957	0.6936	0.002

### Foraminiferal diversity and community composition in SYBH

The alpha diversity of the 12 SYBH samples showed a clear vertical variation. The shallowest sample had systematically the lowest value of the three indexes we used (Observed OTUs, ACE and Shannon; Fig. S1), and the value increased substantially between the samples SYBH2 to SYBH9, although with discrepancies between indexes. We observed a decrease of three indexes in SYBH10 (or SYBH9 for ACE) before another increase of diversity for the samples SYBH11 and SYBH12.

**Figure 5:**
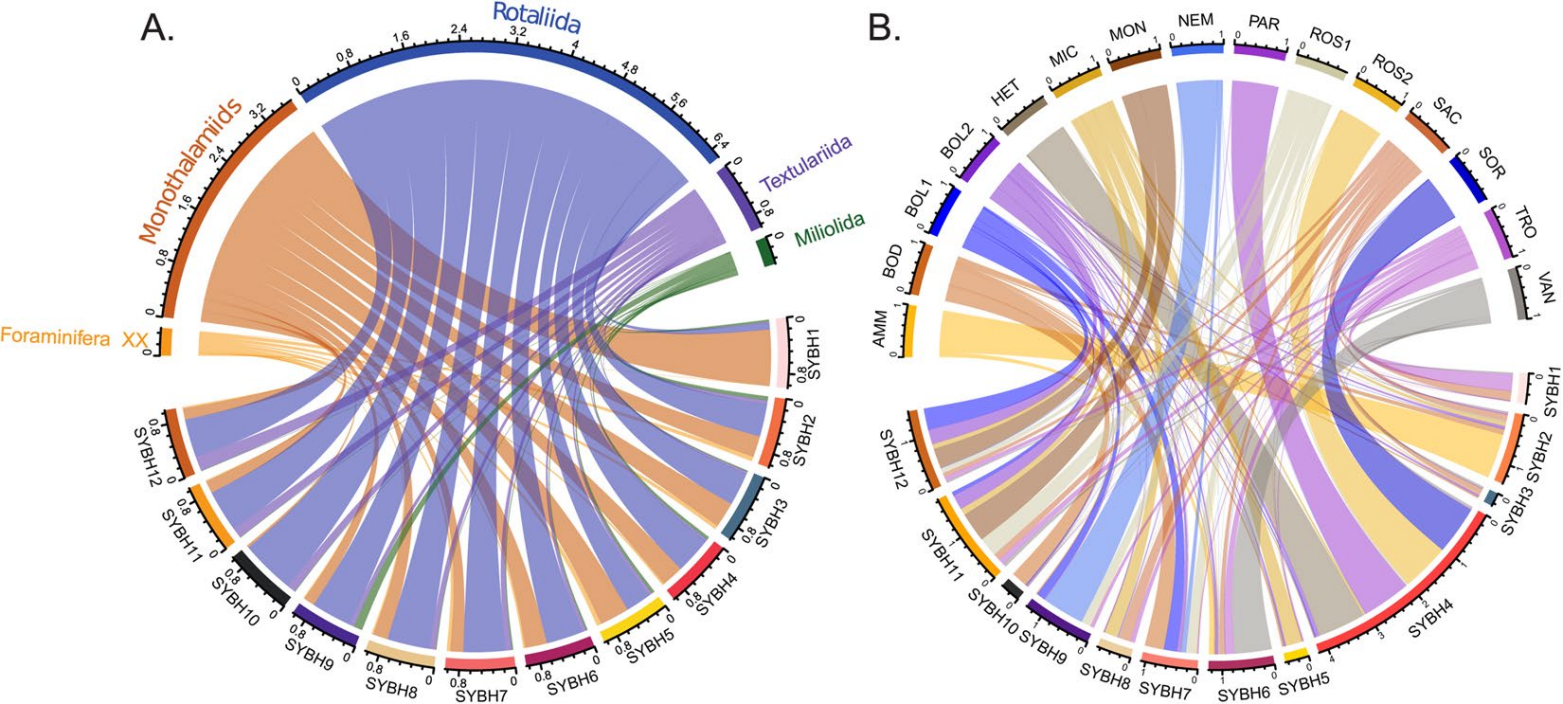
(**A**) Taxonomic composition of foraminiferal assemblages in twelve SYBH sites based on the proportion of reads. (**B**) We picked out all OTUs in SYBH which had an identity percentage with the reference database more than 99% and no less than 10 reads and merged those attributed to the same taxa. As a result, we retained 27 OTUs which were assigned to 15 species: *Ammonia catesbyana* (AMM), *Boderia* sp. (BOD), *Bolivina* sp. (BOL1), *Bolivina variabilis* (BOL2), *Heterostegina depressa* (HET), *Micrometula hyalostriata* (MIC), Monothalamiids_XXX_sp. (MON), *Nemogullmia* sp. (NEM), *Parasorites* sp. (PAR), *Rosalina* sp. (ROS1), *Rosalina vilardeboana* (ROS2), Saccamminidae_X_sp. (SAC), *Sorites* sp. (SOR), *Trochammina hadai* (TRO) and *Vanhoeffenella* sp. (VAN). The proportions of the 15 foraminiferal species in twelve SYBH sites were shown in Fig. 5B which was drawn using the statnet package^61^ and circlize package^62^ based on the number of reads.

This variation of diversity was reflected in the taxonomic composition of foraminiferal assemblages. The shallowest sample SYBH1 (3.1 m) was the only sample dominated by monothalamous foraminifera with 80% of reads (Fig. 5A). The contribution of monothalamiids decreased in the deeper samples in favor to the rotaliids foraminifera. We picked out all OTUs in SYBH which had an identity percentage with the reference database more than 99% and no less than 10 reads and merged those attributed to the same taxa. As a result, we retained 27 OTUs which were assigned to 15 species: *Ammonia catesbyana* (AMM), *Boderia* sp. (BOD), *Bolivina* sp. (BOL1), *Bolivina variabilis* (BOL2), *Heterostegina depressa* (HET), *Micrometula hyalostriata* (MIC), Monothalamiids_XXX_sp. (MON), *Nemogullmia* sp. (NEM), *Parasorites* sp. (PAR), *Rosalina* sp. (ROS1), *Rosalina vilardeboana* (ROS2), Saccamminidae_X_sp. (SAC), *Sorites* sp. (SOR), *Trochammina hadai* (TRO) and *Vanhoeffenella* sp. (VAN) (Fig. 5B). All species showed obvious differences in vertical distribution and environmental preferences. For example, the species AMM mainly distributed in SYBH2, whilst the species SOR, ROS2, PAR and HET mainly distributed in SYBH4. Except for SYBH4, the other nine aerobic sites had smaller total proportions of the 15 species than the two anoxic sites. The proportions of four species: one monothalamiid (MON), one species (ROS1) of genera *Rosalina* and two species (BOL1 and BOL2) of genera *Bolivina* in the anoxic areas (SYBH11 and SYBH12) were higher than their proportions in the other ten sites.

**Figure 6:**
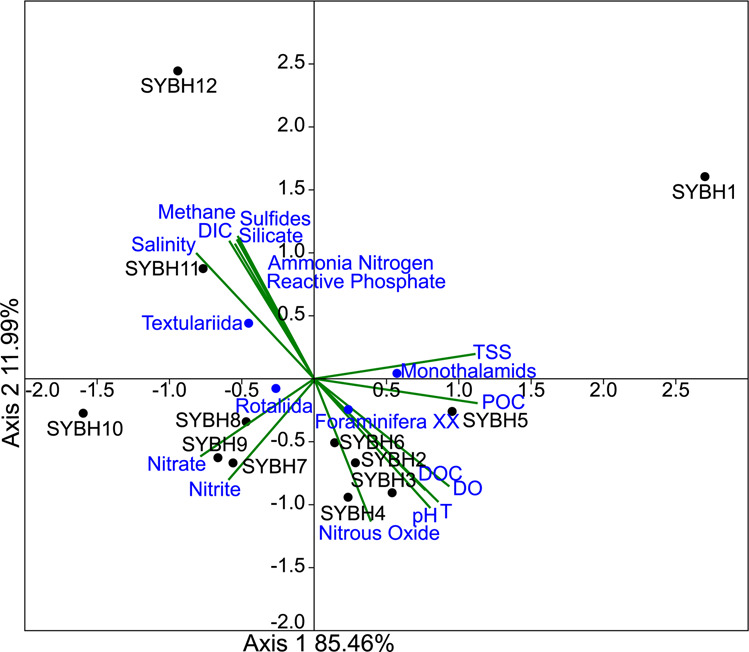
CCA analysis showing the relationship between sixteen hydrochemical factors and relative abundance of four foraminiferal orders in each site of SYBH.

We compared the community composition with sixteen hydro-chemical factors measured in the water column of SYBH (Fig. S2B) recently published^31^. The temperature decreased from 30 °C at the surface to 15 °C at the bottom of the blue hole in two thermoclinic steps between 13 and 20 m and 70 to 150 m whilst the salinity increased from 33.25 to 34.4 between 0 and 150 m. The ammonia nitrogen, DIC (dissolved inorganic carbon), methane, reactive phosphate, silicate and sulfides values were low between the surface and 90 m, increased between 90 and 150 m and their concentrations remained essentially constant below 150 m. Dissolved oxygen (DO) concentration in the upper layer was ~233 μmol L^−1^ and decreased sharply in two steps until 100 m where the water column became anoxic. Concentrations of dissolved organic carbon (DOC), nitrite, particulate organic carbon (POC) and total suspended solid (TSS) had upheavals through the water column but with a general decrease of their absolute values. For nitrous oxide and pH, their concentration decreased with depth, especially from the surface to 100 m. Concentration of nitrate first increased with depth, reached a maximum at 70 m, and then sharply decreased until 100 m (Fig. S2B). The Canonical Correlation Analysis (CCA) analysis (Fig. 6) showed that the twelve samples of SYBH were divided into four groups consistent with those of the Fig. 4B in relationship to the environmental factors. The first group included only SYBH1 due to its singular community dominated by monothalamiids. The second group consisted of five samples, from SYBH2 to SYBH6, collected from 5–20 m and were correlated with POC, DO, DOC, PH and nitrous oxide indicative of near normal environmental conditions. The third group consisted of four samples, from SYBH7 to SYBH10, collected from 20–40 m correlated with nitrate and nitrite. The fourth group included the two samples from the anoxic zone and was correlated with salinity, methane, DIC, sulfides, silicate, ammonia nitrogen and reactive phosphate.

The monothalamous foraminiferal assemblages were negatively correlated with environmental factors such as DIC, reactive phosphate, salinity and silicate, but positively correlated with DO, DOC, POC, pH and temperature. Rotaliida was positively correlated with nitrate, DIC, reactive phosphate, salinity and silicate, and negatively correlated with factors such as DO, DOC, TSS and temperature. Textulariida had a positive correlation with some factors such as DIC, sulfides, salinity, reactive phosphate and silicate, while it had a negative correlation with DO, DOC, pH and temperature (Table 2).

**Table 2:** Spearman’s correlation analysis between the sixteen hydrochemical factors and relative abundance of four foraminiferal orders in SYBH. The r value and *p*-value were separately correlation coefficient and significance. *p*-values beneath 0.05 and 0.01 were marked in a single asterisk and double asterisks, respectively.

Factor		Foraminifera_XX	Monothalamids	Rotaliida	Textulariida
Ammonia nitrogen	r value	0.172	0.175	−0.411	0.028
	*p* value	0.592	0.585	0.185	0.931
DIC	r value	−0.476	−0.846	0.776	0.902
	*p* value	0.117	0.0005**	0.003**	0.00006**
DO	r value	0.414	0.774	−0.771	−0.886
	*p* value	0.181	0.003**	0.003**	0.0001**
DOC	r value	0.249	0.664	−0.734	−0.832
	*p* value	0.436	0.018*	0.007**	0.0008**
Methane	r value	0.086	0.343	−0.550	−0.049
	*p* value	0.791	0.275	0.064	0.880
Nitrate	r value	−0.049	−0.401	0.585	0.134
	*p* value	0.879	0.196	0.046*	0.678
Nitrite	r value	0.056	−0.238	0.510	0.028
	*p* value	0.863	0.457	0.090	0.931
Nitrous oxide	r value	0.063	−0.242	0.504	0.032
	*p* value	0.845	0.449	0.094	0.923
pH	r value	0.406	0.776	−0.762	−0.888
	*p* value	0.190	0.003**	0.004**	0.0001**
POC	r value	0.328	0.577	−0.401	−0.521
	*p* value	0.298	0.049*	0.196	0.082
Reactive phosphate	r value	−0.550	−0.831	0.774	0.887
	*p* value	0.064	0.0008**	0.003**	0.0001**
Silicate	r value	−0.507	−0.848	0.802	0.890
	*p* value	0.092	0.0005**	0.002**	0.0001**
Sulfides	r value	−0.334	−0.382	0.204	0.640
	*p* value	0.289	0.221	0.524	0.025*
Salinity	r value	−0.567	−0.825	0.727	0.867
	*p* value	0.054	0.001**	0.007**	0.0003**
T	r value	0.539	0.811	−0.762	−0.867
	*p* value	0.070	0.001**	0.004**	0.0003**
TSS	r value	0.018	0.513	−0.581	−0.521
	*p* value	0.956	0.088	0.047*	0.083

## Discussion

The Sansha Yongle Blue Hole had the highest level of foraminiferal diversity from the three locations sampled in our study (Fig. 2) and the community structure was highly divergent from the two other locations (Fig. 4). This result is not surprising since the SYBH is located in the pristine environment of the Yongle coral atoll among the Xisha Islands in the South China Sea. The SYBH is geographically close to the Coral Triangle in the Indonesian archipelago that host the richest region in marine diversity^32^. The ocean-wide species richness maps for Large Benthic Foraminifera recently created by Förderer *et al*.^33^ recorded 21 morphotaxa in Xisha Island and a distribution model made by these authors predicted less than 6 morphotaxa in the area close to JZB. It is not possible to make a direct comparison between metabarcoding and morphological diversity assessment because the latter is usually based on size fraction above 125 μm^34^ and therefore do not account for the tiny individuals of the community that may be predominant in the DNA pool. Also, the relative proportions of OTUs observed in the metabarcoding dataset are biased by differential gene copy number that varies between foraminiferal species^35^, as well as the completeness of the reference database and the threshold used to assign OTUs^36^. Although most morphological investigations on foraminifera do not account for the occurrence of the monothalamous species, studies of Gooday *et al*.,^11,37–40^ Goineau *et al*.^41^ and Brandt *et al*.^42^ for bathyal and abyssal areas in the Atlantic Ocean, Indian Ocean, Pacific Ocean and Southern Ocean indicated that monothalamous foraminifera form a large part of meiofaunal communities in deep-sea environments. Metabarcoding studies showed that monothalamous foraminifera are prevalent in nearly all marine environments^19,21,26–29^. We reproduced this observation in JZB and at a lesser extent in the NWP (Fig. 3), but the SYBH displayed a unique structure with a clear dominance of Rotaliida in the assemblages, with the only exception of the shallowest sample where monothalamous foraminifera occupied the highest proportion (Fig. 5A). Unfortunately, we could not sample the reef near the SYBH that could have provided a better point of comparison, but we hypothesize that the dominance of Rotaliida resulted from the abiotic conditions of the SYBH (Fig. 5A), rather than a mere prevalence of Rotaliida in reef ecosystems. This hypothesis is strengthened by the clear structuration of the community in four groups that follows the abiotic gradients (Figs. 4, 6). A recent survey focused on the distribution of the bacterial genus *Vibrio* in the SYBH also showed a clear vertical stratification of the diversity into three groups: aerobic-transition, middle anoxic and bottom anoxic zones^43^, similar to what we observed with foraminifera. In our case, the first group including only one sample (3.1 m, SYBH1) which had the highest concentration of TSS was dominated by the monothalamiids. The second group containing five samples (SYBH2–6) with water depth ranging from 5 to 20 m, where temperature, the concentrations of dissolved oxygen and dissolved organic carbon decreased with water depth, showed a net increase of Rotaliida. The third group including four samples (20–40 m, SYBH7–10) which had higher concentrations of nitrate and nitrite than other samples, was clearly dominated by Rotaliida with a slightly higher proportion of Textulariida than the former two groups. The fourth group (150 m and below, SYBH11–12) was dominated by Rotaliida with the highest proportion of Textulariida among the four groups and it had significantly higher concentrations of ammonia nitrogen, DIC, methane, reactive phosphate, salinity, silicate and sulfide than other groups.

Our results showed that the foraminiferal diversity in two anoxic sites of SYBH was even higher than that in some aerobic sites (Fig. S1), and there were four species: one monothalamiid (MON), one species (ROS1) of genera *Rosalina* and two species (BOL1 and BOL2) of genera *Bolivina* mainly occurring in the anoxic areas of SYBH (Fig. 5B). Interestingly, species of genera *Bolivina* had been shown to be capable of performing denitrification^16^, so we suppose that the two species we observed (BOL1 and BOL2) might also have the denitrification capacity. The above results indicated that there were abundant foraminifera living in the anoxic environments of SYBH, and some foraminiferal species might prefer anoxic conditions to aerobic conditions. Our results are congruent with those of Orsi *et al*.^44^, where transcriptomic analysis of marine sediments collected at the oxic-anoxic transition zone of Namibia showed a high foraminiferal activity and where *Stainforthia* and *Bolivina* were dominated the foraminiferal community. In addition to the *in-situ* observation, a ten-day incubation experiment simulating anoxia showed an increase of foraminiferal gene activity at the onset of the anoxia. Their results suggest that foraminifera significantly increased the level of gene expression under anoxic conditions, which indicated that foraminifera were not only surviving in anoxic environments, but that their activities were stimulated by anoxia.

A key strategy for foraminifera to survive in anoxic environments is to use nitrate as an alternative electron acceptor for denitrification^13–17^. Currently, only a small number of foraminiferal species have been tested for nitrate collection and denitrification capacities, and most studies have focused on the order Rotaliida^16^. Bernhard *et al*.^45^ had reported that one symbiont-bearing monothalamiid could carry out denitrification which was likely catalyzed by the endobionts. The ability of some foraminifera to perform denitrification has been repeatedly verified^13–17^, but their physiological and genetic mechanisms of denitrification are still being explored^45,46^. Bernhard *et al*.^17^ studied the nitrate dynamics in four benthic foraminiferal species with different cellular architecture and microbial endobionts, and their results showed that nitrate reduction could occur in a range of foraminiferal species with or without endobionts, which implied that microbial associates might not solely be responsible for nitrate respiration within the foraminifera. Recently, a study based on large-scale genome and transcriptomes analyses in the foraminiferal genus *Globobulimina* showed a novel denitrification pathway encoded by foraminifera’s own genome^46^. Furthermore, Glock *et al*.^14^ proved that NO_3_^−^ was the preferred electron acceptor in foraminifera from the Peruvian oxygen minimum zone, where the foraminiferal contribution to denitrification was governed by the ratio between NO_3_^−^ and O_2_. Other eukaryotic microbes have been discovered to be able to store nitrate and perform denitrification in anoxic environments as well, such as benthic and pelagic diatom, fungi and ciliate^47^. Even if the survival mechanism of eukaryotic microbes under anoxic conditions is still unclear, we can affirm that most of them have developed physiological strategies to adapt to such environments. Thus, the Sansha Yongle Blue Hole should not be perceived as devoid of life but as treasure trove of diversity and might help scientists to understand how organisms can adapt and thrive under hostile environments.

## Material and Methods

### Study sites and sample collection

We included three entirely different environment of the western Pacific Ocean in our study to compare the foraminiferal taxonomic composition: the shallow coastal Jiaozhou Bay (JZB), the deep-sea abyss of the Northwest Pacific Ocean (NWP) and the Sansha Yongle Blue Hole (SYBH) (Fig. 1). The JZB is a shallow coastal semi-enclosed bay of the Yellow Sea, located on the southern coast of the Shandong Peninsula in the eastern China dominated by clay-silty sand and affected by human activities such as aquaculture and wastewater discharge^48^. Samples were recovered at four sampling sites within the bay between 3.6 and 6.1 m and one sample was recovered at 15.1 m at the entrance of the bay onboard the R/V CHUANGXIN on November 16-17, 2016. A 0.1 m^2^ grab sampler was deployed one time at each site to collect a sediment sample with a thickness of approximately 20 cm. Undisturbed surface 0-1 cm sediments were taken from the grab sampler using a clean spoon and transferred to a sealable polyethylene bag. The sample bags were stored in a portable ice chest and brought back to the laboratory on the day of collection. The sampling area of NWP is located at about 960 km off the eastern coast of Japan, with an average water depth of over 5000 m and is dominated by fine silty clay. The eleven NWP samples were collected from eleven sites between 4080 m and 5830 m using a 0.25 m^2^ modified Gray-O’Hara box corer onboard the R/V KEXUE during February 17 to March 25, 2017. The thickness and volume of the sediment samples were about 0.3 m and 0.075 m^3^, respectively. At each site, all undisturbed surface 0-1 cm sediments were carefully transferred from the box corer to a sealable polyethylene bag and were stored at −80° C on board until further processing.

The SYBH is located within a pristine coral reef of the Yongle coral atoll among the Xisha Islands in the South China Sea. The SYBH is 300 m deep and is shaped like a vertically held ballet shoe with an average width of 130 m at the surface and a minimum width of 26.2 m at the bottom^6,31^. The sediment samples were collected from SYBH along a depth gradient on board R/V CHANGHE OCEAN on May 17–28, 2017 (Fig. 1). Ten samples were recovered between 3.1 and 38.6 m in the oxygenated mixed layer with scuba diving and two samples from anoxic layer were collected at 150 and 300 m respectively with a remotely operated vehicle (ROV). The sediment samples were stored in 50 ml screwcap polypropylene centrifuge tubes and immediately transferred to the liquid nitrogen tank on board until further processing. The supplementary information of the sediment samples is shown in Table S1.

### DNA extraction, PCR amplification and library preparation

Samples of JZB, NWP and SYBH were processed separately to avoid cross-contamination risks, and DNA extraction and PCR amplification were performed in separate rooms. A ca. 0.25 g sediment was taken from the sample to extract the total DNA using the PowerSoil DNA Isolation Kit (Qiagen, Germany). All extraction steps were performed according to the manufacturer’s instructions except for cell lysis. We extended the vortex time from 10 min to 40 min to achieve better cell lysis as described by Lecroq *et al*.^26^. Three replicates of total DNA were extracted from each sediment sample.

In order to identify the successfully extracted samples, we carried out first a series of control PCR reactions, where we amplified the foraminiferal specific fragment together with negative controls at a ratio of 1:1 during extractions and amplification (one blank per sediment sample). The target region of SSU rDNA, consisting of approximately 400 base pairs (bp) was amplified with the foraminiferal-specific primers s14F3 (5′-ACGCAMGTGTGAAACTTG-3′) and s17 (5′-CGGTCACGTTCGTTGC-3′)^9^,^49,50^. Each PCR reaction volume of 25 μL containing 12.5 μL of 2×High-Fidelity PCR Master Mix, 0.5 μL of each primer at 10 μM, 2 μL of DNA template and 9.5 μL of ddH_2_O. The PCR reactions consisted of a pre-denaturation at 94 °C for 90 s, followed by 25 cycles of denaturation at 94 °C for 60 s, annealing at 55 °C for 60 s and extension at 72 °C for 45 s, then immediately followed by additional 10 cycles of denaturation at 94 °C for 30 s, annealing at 55 °C for 30 s and extension at 72 °C for 2 min. These reaction cycles were continuous, and there was no other treatment of the reaction mixture in the process. Each PCR product was mixed with same volume of 1×loading buffer, and electrophoresis was performed on 1% agarose gel for detection and we retained the extract yielding a band of about 400 bp. We repeated the entire procedure of extraction and control PCR for the samples where we initially failed to obtain three positive DNA extracts.

After we obtained positive DNA extraction for each locality, we perform PCR for Illumina sequencing. We repeated the same PCR procedure but using forward and reverse primers tagged with six nucleotide-long sequences appended at their 5′-end to multiplex the PCR products in a unique sequencing library. The triplicated PCR products were pooled on a 2% agarose electrophoresis gel to detect the length of the target band and migrated at 100 volts for 35 min. The mixed PCR products were purified with EZNA TM Gel Etraction Kit (Omega Bio-Tek, Inc, USA) and the concentration of purified PCR products was assessed with Qubit 2.0 Fluorometer (Life Technologies, USA). Sequencing libraries were generated with TruSeq DNA PCR-Free Sample Preparation Kit (Illumina, USA) according to the manufacturer’s protocol and their quality was assessed by the Qubit 2.0 Fluorometer (Life Technologies, USA) and Agilent Bioanalyzer 2100 system^51^. The Illumina HiSeq. 2500 platform was used to sequence libraries and generated 250 bp paired-end reads at the Novogene Bioinformatics Technology Co., Ltd (Beijing). The raw sequence data can be downloaded from the European Nucleotide Archive under BioProject PRJEB35877 (https://www.ebi.ac.uk/ena/data/view/PRJEB35877).

### Data quality control and processing

Raw paired-end reads were raw data obtained using the Illumina HiSeq sequencing platform. They were de-multiplexed to samples based on their unique barcode sequences. The maximum number of errors in barcode was 1.5. We discarded unqualified reads, and truncated the remaining reads by cutting off the barcode and primer sequences. Raw paired-end reads were merged using FLASH (V1.2.7)^52^, and the spliced sequences were called raw reads. Quality filtering on the raw reads were performed according to the QIIME (V1.9.1)^53^ quality-controlled process. The following raw reads were discarded: (i) raw reads with adapter contamination (>10 nt aligned to the adapter); (ii) raw reads with ≥10% unidentified nucleotides (N); (iii) raw reads with consecutive high-quality bases (≥Q20) less than 75%; (iv) raw reads with low-quality bases (≤Q5) more than 50%. At last, detected and removed the chimera sequences. After filtering out low-quality sequences and chimeras, we obtained the high-quality effective reads^54^. Then we performed dereplication of the high-quality effective reads to find unique sequences (also called “unique-identical sequences”). During this step, the fastx_uniques command (minuniquesize = 2) was used to find the set of unique sequences and add size annotations to them. After dereplication, singletons which appeared only once in the complete dataset were removed and the unique sequences were sorted by decreasing abundance. We used UNOISE3 denoising algorithm in USEARCH (v11.0.667)^55^ to identify correct biological sequences in the unique sequences. Most of the low-abundance sequences are usually noisy. Therefore, we set the minimum abundance size to ten in this step and discarded the unique sequences with lower abundances to reduce false-positive errors in the reserved biological sequences. After dereplication and denoising, we obtained the representative sequences of OTUs. We used the usearch_global command in USEARCH (v11.0.667)^55^ to align all high-quality effective reads to the representative sequences of OTUs with 97% identity threshold and calculated the abundance of OTUs. For taxonomic annotations of OTUs, the representative sequences of OTUs were compared to the PR^2^ database (v4.11.1)^30^ using BLAST (v2.7.1). The closest hit which might not be the actual taxon was retained for each OTU. Prior to annotation, we updated the taxonomy of benthic foraminifera based on the recent classification^56^ of rotaliid by modifying the taxonomic path of the foraminifera sequences to integrate the names of the new super-families (Table S4). As a final step in our curation process, we excluded the OTUs that could not be assigned to Foraminifera and the OTUs with an abundance of less than 10 reads or that were occurring in a single sample.

### Statistical analysis

We calculated the OTUs rarefaction curves at the regional and sample level based on the number of eventually retained OTUs and reads using the iNEXT package^57^ and normalized the data of each sample using a standard of reads number corresponding to the sample with the least reads (41,146) before calculating four alpha diversity indexes with QIIME (V1.9.1)^53^. The results of both analyses are displayed on Fig. 2 by using the package ggplot2^58^. Proportions of OTUs and reads assigned to different taxonomic groups in three locations were visualized in Fig. 3 with the ggplot2 package^58^. Based on the normalized data, we analyzed the community composition by calculating a Non-Metric Multi-Dimensional Scaling on Bray-Curtis similarity coefficient using the vegan package^59^ (Fig. 4A) and a heatmap based on the Spearman’s correlation with pheatmap package^60^ (Fig. 4B). We used the statnet^61^ and circlize^62^ packages to draw the taxonomic composition of foraminiferal assemblages in twelve SYBH samples (Fig. 5). Finally, we analyzed the relationship between the occurrence of the foraminifera and the abiotic factors measured in the SYBH by Xie *et al*.^31^ with a CCA (Fig. 6) and Spearman’s correlation analysis (Table 2) using Past (v3.25)^63^.

## Supplementary information

Supplementary Information.

Supplementary Information.

Supplementary Information.

Supplementary Information.

Supplementary Information.

Supplementary Information.

## Data Availability

The raw sequence data can be downloaded from the European Nucleotide Archive under BioProject PRJEB35877 (https://www.ebi.ac.uk/ena/data/view/PRJEB35877).
