# Supplementary figures and images for: Diversity hotspot and unique community structure of foraminifera in the world’s deepest marine blue hole – Sansha Yongle Blue Hole
Source: Sci Rep. 2020 Jun 24;10:10257. doi: 10.1038/s41598-020-67221-0 (PMC7314809; doi:10.1038/s41598-020-67221-0)

Indexes

Observed OTUs

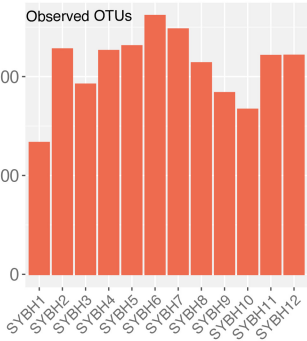

ACE

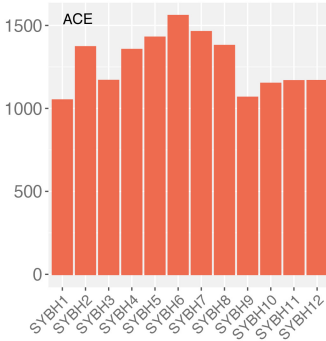

Shannon

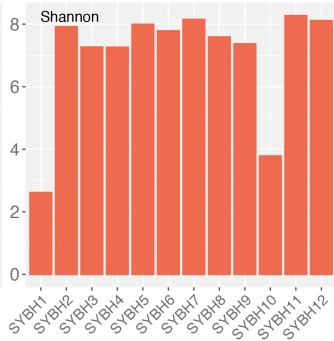

Sites

Supplement: Supplementary file 1 — Supplementary Information. [file 41598_2020_67221_MOESM1_ESM.pdf]

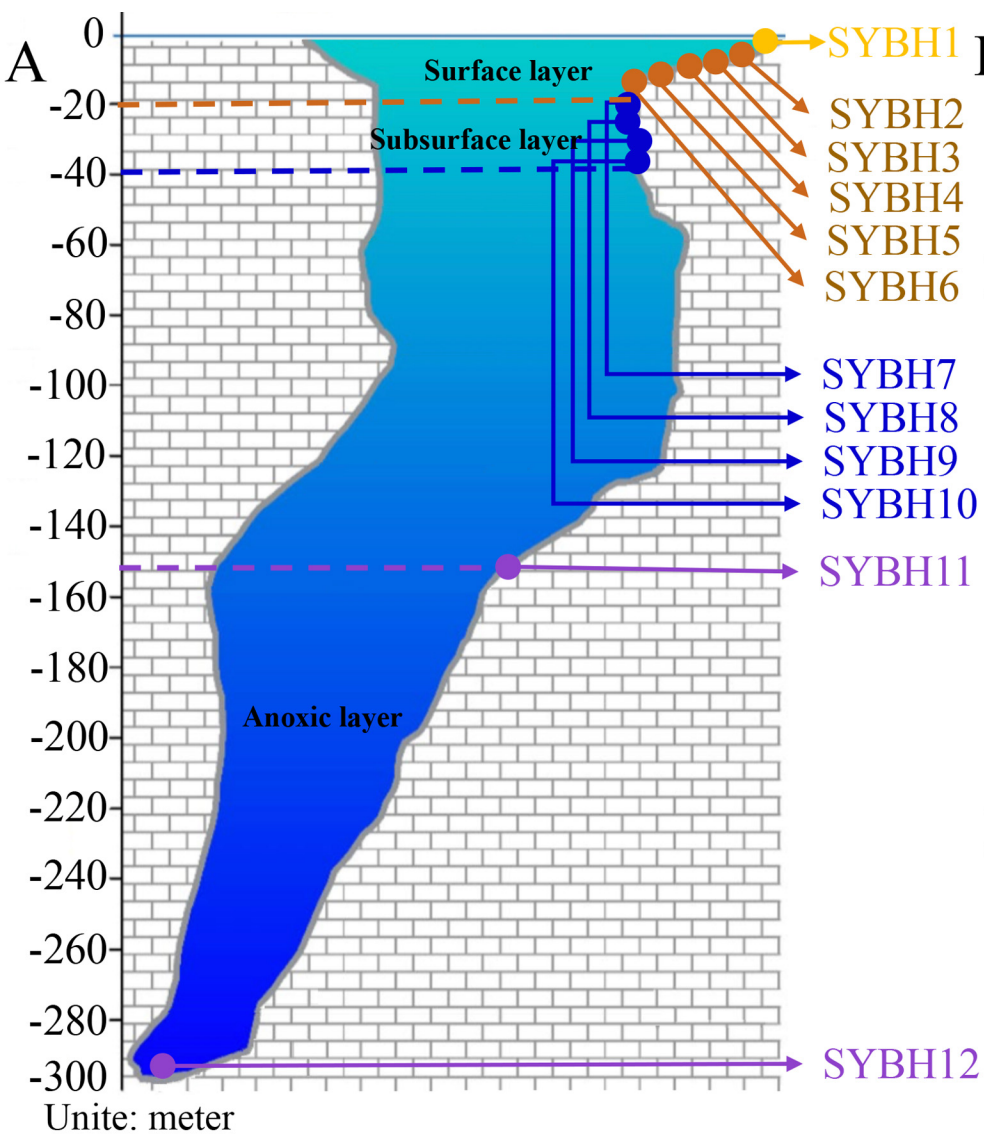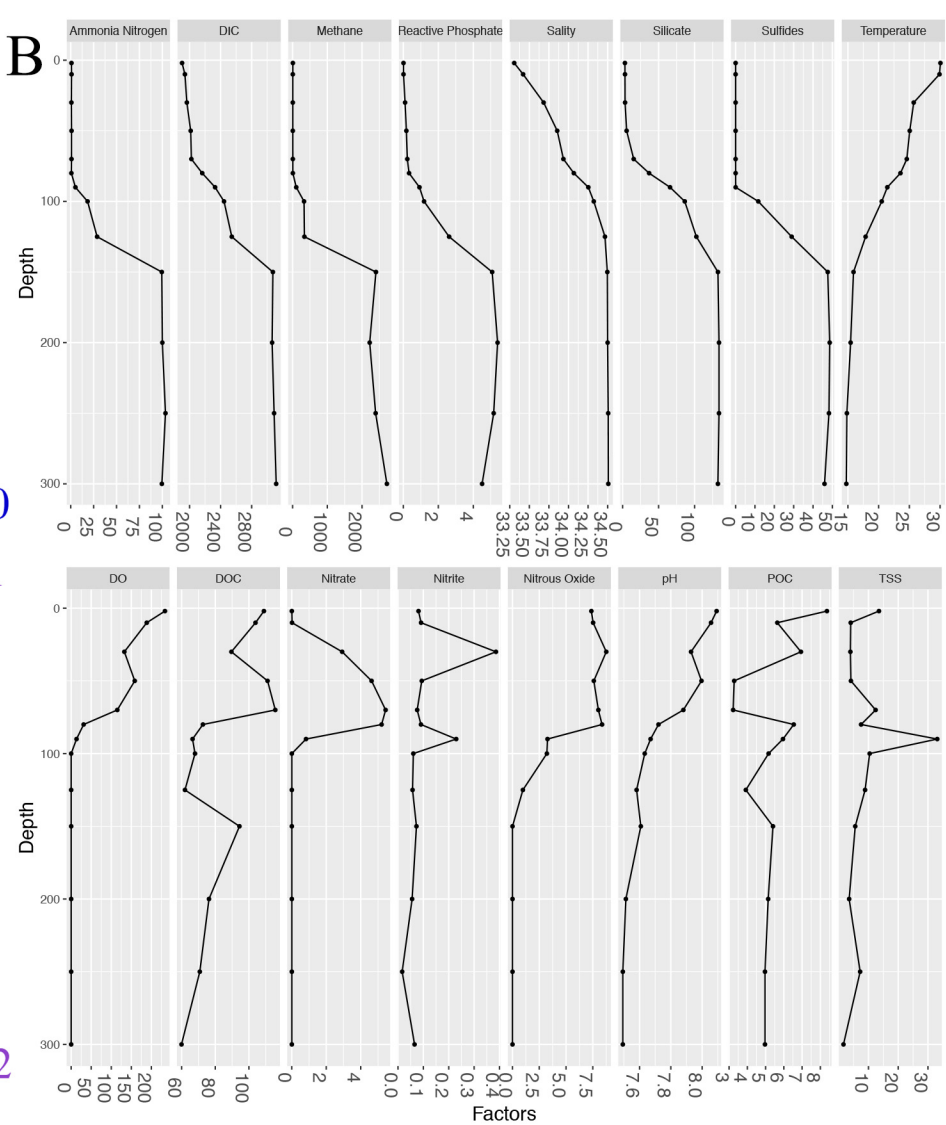

Supplement: Supplementary file 2 — Supplementary Information. [file 41598_2020_67221_MOESM2_ESM.pdf]
